# RNA-seq of *Ranunculus sceleratus* and Identification of Orthologous Genes among Four *Ranunculus* Species

**DOI:** 10.3389/fpls.2016.00732

**Published:** 2016-05-31

**Authors:** Shu-Ying Zhao, Ling-Yun Chen, Yan-Li Wei, Qing-Feng Wang, Michael L. Moody

**Affiliations:** ^1^Key Laboratory of Aquatic Botany and Watershed Ecology, Wuhan Botanical Garden, Chinese Academy of SciencesWuhan, China; ^2^Beijing Genome Institute-Shenzhen, Beishan Industrial ZoneShenzhen, China; ^3^Department of Biological Sciences, University of Texas at El PasoEl Paso, TX, USA

**Keywords:** transcriptome, *Ranunculus*, orthologous genes

## Introduction

*Ranunculus* L. is an early diverging clade in the Angiosperm phylogeny. The split between terrestrial and aquatic/semi-aquatic lineages within the genus occurred within 20 Ma (Emadzade and Horandl, 2011), which is much younger than for some well-known aquatic lineages such as *Ceratophyllum* and *Potamogeton*. *Ranunculus sceleratus* Linn. is a semi-aquatic plant commonly found in paddy fields, streams and lakes in Asia, Europe and North America (eFlora of China, http://www.efloras.org/). The plant has also been studied for its unique toxicological and pharmacological properties (Prieto et al., 2003). Its karyotype is 2*n* = 32, *x* = 8 in mainland China (Yang, 2000). It usually roots in inundated soils, with stems and leaves emergent. The semi-aquatic *R. sceleratus* has been hypothesized to be an inter-mediate type in the transition from a terrestrial to aquatic habitat within the genus (Barrett et al., 1993; Prieto et al., 2003).

In this study, we generated RNA-seq data of *R. sceleratus* and analyzed this in relation to the RNA-seq data of *Ranunculus bungei* Steud., *Ranunculus cantoniensis* DC., and *Ranunculus brotherusii* Freyn (Chen et al., 2015). Our aim was to generate RNA-seq data of *R. sceleratus* and identify the orthologous genes among the four species.

### Links to deposited data

RNA-seq data of *R. sceleratus* was deposited at NCBI sequence Reads Archive (no. SRP072329). It is in SRA format. ITS sequence of *R. sceleratus* was deposited in GenBank (no. KT957621). All the unigenes of *R. sceleratus*, data matrices of ITS and chloroplast DNA, sequences of the 3455 putative orthologous clusters identified using the program OrthoMCL, and 884 clusters after filters were deposited in figshare (https://figshare.com/s/f40d8f9bd8f894f2d630; sequences in fasta format). These data can be used for further transcriptome assembly, selective pressure estimation, phylogenetic analyses, etc.

## Materials and methods

### Plant material, sequencing, and *de novo* assembly

*R. sceleratus* was collected from East Lake (30°32′44.97″ N, 114°42′10.07″ E), Hubei, China, in Oct. 2014. Living plant material was brought to laboratory within 20 min. Leaves, stems, and roots from one individual were immediately sampled and frozen with liquid nitrogen. RNA extraction using RNAisoTM Plus (Takara, Qingdao, China) and quality checking were performed using the methodology in Chen et al. (2015). The RNA sample was transported to a laboratory of Beijing Genomics Institute (BGI) in Wuhan. mRNA was extracted using oligo (dT) magnetic beads, followed by breaking into small fragments. A cDNA library with inserted size c. 200 bp was constructed. Paired-end sequencing (2 × 90 bp) was performed using an Illumina HiSeq™ 2000 sequencer.

Raw reads were processed using Filter_fq (an internal program of BGI) to remove adaptor sequences, reads with unknown base calls (*N*) larger than 5%, and reads with low quality bases (quality value ≤ 10) more than 20%. *De novo* assembly using the clean reads was performed using Trinity v. 20130225 (Grabherr et al., 2011) with parameters—min_contig_length 100,—group_pairs_distance 250,—path_reinforcement_distance 85, and default parameters. Unigenes recovered from Trinity were clustered to get long sequence unigenes and remove redundancies using program TGI Clustering Tool (TGICL) v. 2.1 (Pertea et al., 2003) with parameter -l 40, -c 10, and -v 20.

The RNA-seq data of *R. bungei, R. cantoniensis*, and *R. brotherusii* (NCBI Sequence Read Archive, no. SRR1822558, SRR1822529, SRR173752s) were also used.

### Nuclear ITS and chloroplast DNA sequences

(1) The internal transcribed spacer regions (ITS1, ITS2) and 5.8S ribosomal RNA gene for *R. sceleratus* was generated with the methodology in Chen et al. (2015); ITS for *R. bungei, R. cantoniensis*, and *R. brotherusii* were accessed from GenBank (nos. KP336399, KP336398, KP336400). Close relative of *Ranunculus*, viz. *Laccopetalum giganteum* and *Krapfia clypeata* (GenBank nos. GU552271, GU552272), were used as outgroups following Emadzade and Horandl (2011). (2) cpDNA sequences for the four *Ranunculus* species were extracted from non- redundant unigenes of the four species; the chloroplast genome of *Clematis terniflora* was accessed (GenBank no. NC_028000). *C. terniflora* was used as an outgroup as it is the closest relative of *Ranunculus* with a complete chloroplast genome available in GenBank. The DNA alignment was conducted using Mauve v. 20150226 (Darling et al., 2004). Conserved sequences for the five species were concatenated and yielded a matrix with 29,654 bp length for each species. Maximum likelihood (ML) analyses were conducted for ITS and cpDNA datasets separately using RAxML v. 8.1.20 (Stamatakis, 2006) with the GTRGAMMA model and fast bootstrap (1000 replicates) analyses. Phylogenetic relationships of the four *Ranunculus* species recovered from ITS and cpDNA are congruent with each other. ITS phylogeny: [(*L. giganteum*, K. *clypeata*), (*R. cantoniensis*, (*R. brotherusii*, (*R. bungei, R. sceleratus*)71)100)85); cpDNA phylogeny: (*C. terniflora*, (*R. cantoniensis*, (*R. brotherusii*, (*R. bungei, R. sceleratus*)100)100)].

### Identification of orthologous genes

Step by step strategies were adopted to find orthologs and exclude possible paralogs:

(1) Orthologous clusters among *R. bungei, R. sceleratus, R. cantoniensis, R. brotherusii*, and *Vitis vinifera* were identified using OrthoMCL (Li et al., 2003) with default settings according to Wissler et al. (2011). More species may increase efficiency in computational screening for orthologs. Protein sequences of *V. vinifera*, which show high similarity to *Ranunculus* species, were downloaded from Genoscope (http://www.genoscope.cns.fr/spip/). Clusters with ≥1 sequence per species were used. If >1 sequence of any species was included in a cluster, only the sequence showing the highest similarity to sequences of other species was kept. (2) After removing the sequences of *V. vinifera*, orthologous clusters were aligned by MUSCLE (Edgar, 2004) with default parameters. Clusters with alignment length < 200 bps and including unexpected stop codons were discarded. (3) Saturation tests were performed to remove orthologs saturated at synonymous sites. The third codon positions of each ortholog were extracted, then used to estimate branch lengths of the gene tree with the general time reversible model in the PAML package v. 4.8 (Yang, 2007). The cluster was discarded from further analyses if branch length of one or more branches was < 1.

(4) ML analyses were also conducted for all the clusters from the last step using the same methods as for the nuclear ITS and chloroplast DNA sequence data sets, but without the outgroup setting. The clusters, which had different species relationships than the ITS and cpDNA trees, were excluded from further analyses. This method is able to exclude the possible paralogs (Zeng et al., 2014). (5) A local blast database was constructed using protein sequences accessed from NCBI (Aug. 2015) with the software package NCBI BLAST+ v. 2.2.31. Protein sequences of 19 species, which showed high similarity to sequences of *Ranunculus* in our preliminary analyses, were incorporated, viz. *Amborella trichopoda, Arabidopsis thaliana, Brassica napus, Camelina sativa, Citrus sinensis, Cucumis sativus, Elaeis guineensis, Fragaria vesca, Glycine max, Gossypium raimondii, Malus domestica, Medicago truncatula, Nelumbo nucifera, Populus trichocarpa, Prunus mume, Ricinus communis, Sesamum indicum, V. vinifera*, and *Zea mays*. In total, 752,004 sequences were incorporated. All the orthologs from the last step were annotated to the local database using BLASTX with *E* = 10^−3^ and default settings. If any of the four orthologs within a cluster were matched to different sequences, the cluster was excluded from further analyses using perl scripts. (6) The aligned clusters were inspected by eye in BioEdit v. 7.2.5 (Hall, 1999). Alignment problems at either end of sequences were deleted, and clusters with problems in the middle were excluded.

## Results

### *De novo* assembly

We generated 46.7 million of clean paired reads totaling 8.4 × 10^9^ bp of RNA-seq data for *R. sceleratus*. *De novo* assembly yielded 111,101 contigs, with total length 37,271,711 bp, mean length of 335 bp and N50 at 635 bp. TGICL yielded 61,321 non-redundant unigenes, with total length of 37,881,109 bp, mean length of 618 bp, and N50 at 1054 bp (Table 1).

**Table 1:** **Transcriptome statistics of *Ranunculus sceleratus***.

**Attributes**	**Values**
**PRE-ASSEMBLY**	
NCBI bioproject ID	PRJNA315806
NCBI biosample ID	SAMN04570362
NCBI SRA ID	SRP072329
Platform	Illumina hiseq2000
Read length	90
Number of paired-end reads	46,672,970
Number of total bases	8,401,134,600
**POST-ASSEMBLY**	
Number of non-redundant unigenes	61,321
Number of total bases	37,881,109
N50	1054

### Orthologous clusters

A total of 3455 putative orthologs were recovered for *R. sceleratus, R. bungei, R. cantoniensis*, and *R. brotherusii* using OrthoMCL. After removing the clusters with aligned length < 200 bp, including unexpected stop codons, and saturation at synonymous sites, 2637 orthologous clusters were retained. After removing the clusters that were not consistent with phylogeny, blast or alignment, 884 clusters were retained.

## Author contributions

SZ and QW conceived this study; SZ and LC carried out experimental works and with MM drafted the manuscript; SZ, LC, YW and ZH performed data analyses. All authors gave approval of this manuscript to be published.

## Funding

This research was supported by grants from the National Natural Science Foundation of China (no. 31500192), the Chinese Academy of Sciences (no. XDAO5090305), and the Special Foundation for State Basic Working Program of China (no. 2013FY112300).

### Conflict of interest statement

The authors declare that the research was conducted in the absence of any commercial or financial relationships that could be construed as a potential conflict of interest. The reviewer AC and handling Editor AM declared their shared affiliation, and the handling Editor states that the process nevertheless met the standards of a fair and objective review.
